# Embryogenesis of esophageal atresia: Is localized vascular accident a factor?

**DOI:** 10.4103/0971-9261.55158

**Published:** 2009

**Authors:** Hemonta K. Dutta, Shree Harsh

**Affiliations:** Department of Pediatric surgery, Assam Medical college, Dibrugarh, Assam, India

**Keywords:** Embryogenesis, esophageal atresia

## Abstract

Several theories on embryogenesis of esophageal atresia have been proposed, none could explain the whole spectrum of this anomaly. We report a new variant of esophageal atresia in which the two blind pouches were joined by an atretic band. Histology of the atretic part showed groups of striated muscle arranged haphazardly without any lumen. The existing theories on etiology of esophageal atresia cannot explain this variant. However, localized vascular accident during intrauterine life resulting in disturbances in regional microcirculation could be a possible factor as demonstrated by Louw and Barnard in relation to jejunoileal atresia. This is contrary to the current understanding that disproportionate growth of the horizontal esophageal folds results in esophageal atresia.

## INTRODUCTION

The embryogenesis of esophageal atresia (EA) is poorly understood. Theories such as intra embryonic pressure, vascular accidents, failure of recanalization, disproportionate growth of the lateral epithelial folds etc. were inadequate to explain the whole spectrum of this malformation. The latest theory based on electron microscopic studies suggests that overgrowth of a dorsal horizontal fold in the region of the tracheoesophageal separation would result in EA and tracheoesophageal fistula (TEF).[1] This theory also proposes that late ischemia of an already formed esophagus could result in pure EA.

## CASE REPORT

**Figure 1:**
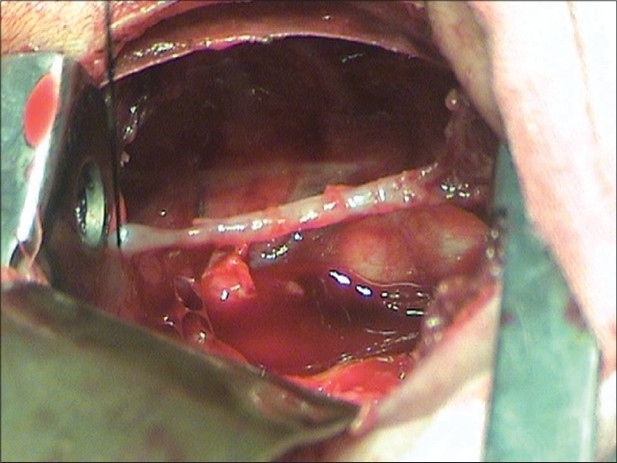
Atretic band

**Figure 2:**
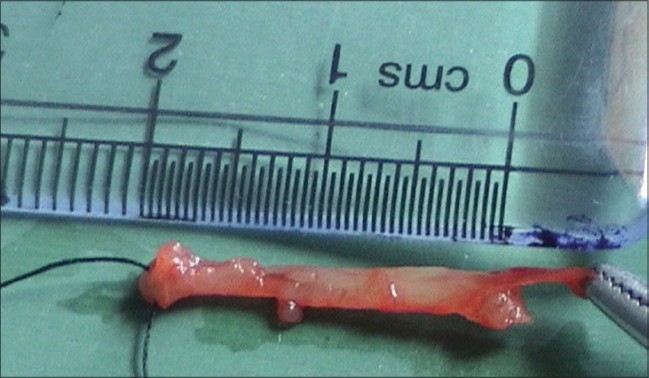
Excised band

**Figure 3:**
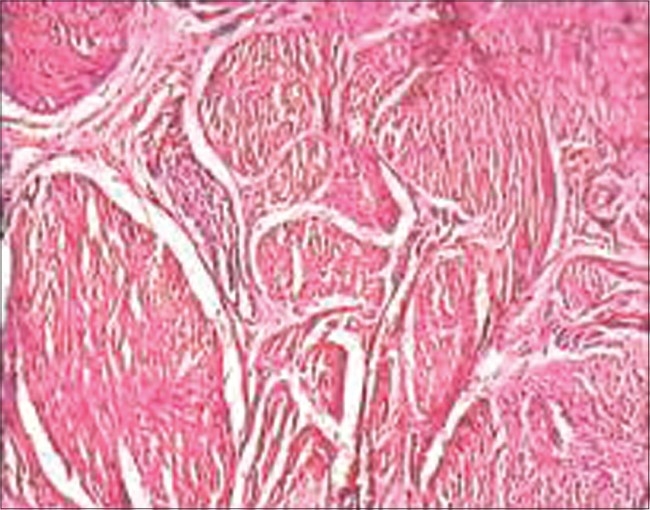
Histology of atretic part shows disorganized striated muscle

The author observed a new variant in a patient with pure esophageal atresia. A two-day old male baby weighing 2.6 kg presented with frothing from mouth, high respiratory rate and a flat abdomen. A catheter through the mouth did not go beyond 10 cm from the lower jaw. Water soluble contrast study showed a blind upper pouch. A right posterolateral thoracotomy was done. It was observed that both the blind ends were joined by an atretic segment about 24 mm long and two mm thick [Figures 1 and 2]. The segment seemed to have a few muscle fibers [Figure 3]. The atretic segment was excised, blind ends opened and a primary anastomosis performed. The patient had an uneventful recovery and was discharged on 12^th^ post operative day.

## DISCUSSION

The esophagus can be identified at three weeks of gestation. In the subsequent two weeks, elongation of the esophagus occurs mainly due to ascent of the larynx. Ventral tracheal primordium develops at this stage. Rosenthal proposed that this process involves apposition of two lateral longitudinal epithelial folds.[2] Failure of this apposition leads to tracheoesophageal fistula formation while apposition too posteriorly results in EA. Smith proposed that during the rapid phase of esophageal elongation, over thinning of the esophagus results in EA.[3] Tandler's theory of failure of recanalization resulting in atresia may explain the rare congenital esophageal web, but fail to explain the full thickness defect in EA.[4] The presence of abnormal branchial vessels seen in some cases of EA suggest a coincident rather than causal relationship.[5,6] Based on detailed saggital sections and scanning electronic microscopic studies on chick embryos, Kluth and Habenicht proposed the theory of esophagotracheal separation by dorsal and ventral horizontal folds.[7] Overgrowth of the dorsal fold results in distal fistulas continuity with the trachea. Although this theory comes close to explaining many of the types of EA and TEF, it fails to explain embryogenesis of some variant of EA. Variants of esophageal atresia, reported in literature, such as esophageal atresia with distal tracheoantral fistula associated with congenital intrathoracic stomach and situs inversus (SI),[8] esophageal atresia with triple fistula,[9] esophageal atresia with an abnormally long lower segment that entered the trachea in the region of the upper thoracic aperture, a long atretic proximal portion of the lower segment, and an additional membrane in the lower segment,[10] esophageal atresia associated with esophageal heterotopic pancreas[11] etc. could not be explained on the basis of these theories. There are still other variants such as pure esophageal atresia that showed spontaneous recanalization,[12] which could be the subtype II5 of type 2 in Kluth's atlas of esophageal atresia with 2 blind esophageal ends and a cyst occupying the intervening space.

Sinha *et al*. reported another variant with a TEF between the lower pouch and trachea, with a cystic dilatation in the midportion.[13] The tracheal end of the fistula was obstructed by a membranous septum at both ends of a cystic dilatation, leading to a diagnosis of pure EA (gasless abdomen). In the present variant, which was not reported in the literature earlier, two blind ends of the esophagus are connected by an atretic band. The band was 24 mm long and histology of the part showed presence of disorganized striated muscle groups without any lumen. This is similar to type II atresia of the small bowel. Etiology of this variant could be explained on the basis of the antenatal vascular accident theory proposed by Louw in jejunoileal atresia.[14] Through their classic experimental study in 1955, Louw and Barnard demonstrated that intrauterine vascular insult to the developing bowel results in variable degree of atresia.[2] This study was further supported by some other experimental studies.[15–17] Similar observations noted in humans led to wide acceptance of this theory.[18–20] Jejunoileal atresia, in rare instances, has been found to be associated with esophageal, gastric, duodenal and colonic atresias,[18–20] which may point to a common etiological factor.
